# Effect of De-Twinning on Tensile Strength of Nano-Twinned Cu Films

**DOI:** 10.3390/nano11071630

**Published:** 2021-06-22

**Authors:** Chia-Hung Lee, Erh-Ju Lin, Jyun-Yang Wang, Yi-Xuan Lin, Chen-Yu Wu, Chung-Yu Chiu, Ching-Yu Yeh, Bo-Rong Huang, Kuan-Lin Fu, Cheng-Yi Liu

**Affiliations:** Department of Chemical Engineering and Materials Engineering, National Central University, Jhong-Li 32001, Taiwan; chiahunglee840213@gmail.com (C.-H.L.); 609mua@gmail.com (E.-J.L.); nicky821021@gmail.com (J.-Y.W.); 611cheryl@gmail.com (Y.-X.L.); devil84425@gmail.com (C.-Y.W.); cliffabb559@gmail.com (C.-Y.C.); gina80085@gmail.com (C.-Y.Y.); yui741@gmail.com (B.-R.H.); zj4810513@gmail.com (K.-L.F.)

**Keywords:** de-twinning, nano-twinned Cu, tensile strength

## Abstract

Tensile tests were carried on the electroplated Cu films with various densities of twin grain boundary. With TEM images and a selected area diffraction pattern, nano-twinned structure can be observed and defined in the electroplated Cu films. The density of the nano-twin grain structure can be manipulated with the concentration of gelatin in the Cu-sulfate electrolyte solution. We found that the strength of the Cu films is highly related to the twin-boundary density. The Cu film with a greater twin-boundary density has a larger fracture strength than the Cu film with a lesser twin-boundary density. After tensile tests, necking phenomenon (about 20 μm) occurred in the fractured Cu films. Moreover, by focused ion beam (FIB) cross-sectional analysis, the de-twinning can be observed in the region where necking begins. Thus, we believe that the de-twinning of the nano-twinned structure initiates the plastic deformation of the nano-twinned Cu films. Furthermore, with the analysis of the TEM images on the nano-twinned structure in the necking region of the fractured Cu films, the de-twinning mechanism attributes to two processes: (1) the ledge formation by the engagement of the dislocations with the twin boundaries and (2) the collapse of the ledges with the opposite twin-boundaries. In conclusion, the plastic deformation of nano-twinned Cu films is governed by the de-twinning of the nano-twinned structure. Moreover, the fracture strength of the nano-twinned Cu films is proportional to the twin-boundaries density.

## 1. Introduction

Owing to its coherence feature, the twin boundary has the smallest grain-boundary energy among all types of grain boundaries. For FCC Cu, the low stacking fault energy (~22 mJ/m^2^) easily results in the nano-twinned structure in the Cu phase [1,2,3,4]. It has been reported that, with the high density of twin boundary, the strength, ductility, and conductivity of the Cu can be enhanced [5,6,7,8,9,10,11]. Thus, recently, the Cu conduction lines produced with a high density of the twin boundaries have attracted great attention due to its unique properties in the conductivity and electro-migration resistance [12,13,14]. Twin boundaries in the Cu phase can be produced by either mechanical or annealing treatment, which are called mechanical twin and annealing twins, respectively [15,16,17,18,19,20]. Furthermore, many researchers reported that twin boundaries can be produced by means of the electroplating [9,12,21,22,23]. The amount and density of the twin boundaries can be manipulated by the specific additives, pulse current modes, and different types of electroplating solution [9,24,25,26]. Typically, two kinds of twin grains can be produced in the electroplated Cu films: the equi-axised twin grains (typically dispersing in the Cu matrix) and columnar twin grains (the Cu columns grow perpendicularly to the plating substrate) [27,28,29,30].

Like other grain boundaries, twin boundary can increase the strength of metals by retarding the motion of the dislocations driven by the external stress. For example, Li et al. have compared the mechanical of the Cu films with the twin granular structure mentioned above [21]. However, many researchers also have found that the twin boundaries could be removed by imposing the external stress, which is called de-twining [31,32,33,34]. The above statement implies that, under the external stress, the twin boundary would retard the dislocation and also the twin boundary would be de-twinned by the external stress. Hence, it is very important to understand, during the tensile testing, how de-twinning occurs and how it plays a role in affecting the fracture strength. In this work, the electroplated Cu films were prepared with the commercial electroplating solution (virgin makeup solution) added with various concentrations of gelatin. The electroplated Cu foils consist of Cu columnar grains, which grew normal to the plating substrate. Nano-twin grains can be observed in the Cu columnar grains in the electroplated Cu films. Firstly, after tensile tests, de-twinning can be observed by focused ion beam (FIB) cross-sectional analysis on the fractured Cu foils. The de-twinning mechanism can be studied and proposed. Then, the de-twining condition can be related to the measured fracture strength. Moreover, with varying concentration of gelatin, the density of twin boundary in Cu columnar grains can be manipulated. Therefore, by varying the density of twin boundary in Cu columnar grains, the effect of the density of twin boundary on the de-twining process can be studied. This effect is further related to the mechanical strength of the electroplated Cu foils. The goal of this work is to investigate the interaction effect between the dislocation restriction and the de-twining of the twin boundaries with the external stress.

## 2. Materials and Methods

Cu films (thickness about 18 μm) were electroplated on the 10 mm × 10 mm Al substrates. The setup of the electroplating is described as following. The anode is the pure Cu sphere ball (purity 99.9%). Before the Cu plating process, the anode Cu sphere ball was polished with the sandpaper (120 grits) to remove the possible surface oxide and contamination. Furthermore, the surface of the cathode Al substrate was polished with the sandpapers (2500 and 4000 grits), and then, cleaned by the solvents, i.e., acetone and ethanol. The distance between the anode Cu ball and the cathode Al substrates was maintained about 50 mm in the process of Cu electrodeposition. A constant current density 0.08 A/cm^2^ is provided by DC power supply (PST-3202, Gwinstek, New Taipei City, Taiwan). The cathode Al substrate was rotated with 220 rpm at room temperature (298 K). The basic Cu electrolyte provided from WESI International Inc. (Taoyuan, Taiwan) was used for the Cu electroplating process. The plating time is 10 min, and the Cu film thickness is maintained at about 20 μm. After the electroplating process is completed, the Cu films can be easily detached from the Al substrate easily because of the weakness of the adhesion between the Cu film and Al substrate. The major components in the Cu electrolyte are composed of CuSO_4_, H_2_SO_4_, and Cl^−^. Using the Cu-sulfate electrolyte solution as the base solution, different concentrations of gelatin was added to produce the columnar nano-twinned Cu (denoted as nt-Cu throughout this paper) films with different twin boundary density. Based on our preliminary study, the best gelatin concentration for producing the twinning microstructure in the Cu film is in the range of 7.8 to 8 g/L. Any gelatin concentration outside this range would have fine Cu grains (not Cu twin grains) in the Cu film. Thus, the adding concentrations of gelatin additives are chosen as (8 g/L), (7.9 g/L), and (7.8 g/L).

After Cu electroplating, the nt-Cu films were stripped off from the Al cathode substrates. The surface roughness was measured by Alpha-stepper measurement. The surface roughness (Ra value) of the nt-Cu plated with gelatin concentration (8 g/L), (7.9 g/L), and (7.8 g/L) are 276.0, 352.5, and 369.4, respectively. The TEM images and selected area diffraction analysis by HR-TEM (bright field image; JEOL JEM-2100, Tokyo, Japan) were used to analyze the twin structure of the electroplated nt-Cu films. The crystalline orientation of the microstructure of the electroplated nt-Cu films is determined by the TEM selected area diffraction analysis. The detail microstructure of the nt-Cu films was observed by the cross-sectional images with a focused ion beam (FIB). With the FIB cross-sectional images, the twin spacing of the lamellar grains and twin boundary density can be calculated. Uniaxial tensile tests were performed on the electroplated nt-Cu films at a strain rate of 5 × 10^−3^ s^−1^ at room temperature. The tensile stress was parallel to the electroplated nt-Cu films. The stress–strain curves can be obtained from the tensile tests. Tensile tests were carried out at room temperature with the strain rate of 5 × 10^−3^ s^−1^. Then, the mechanical properties, such as, Young’s modulus, yield strength, fracture strength, and elongation to failure can be defined from the stress–strain curves of the tested nt-Cu films. Moreover, after tensile tests, again, FIB cross-sectional images were used to investigate the detail deformation microstructure of the tested nt-Cu films.

## 3. Results

### 3.1. Microstructure of nt-Cu Films

**Figure 1:**
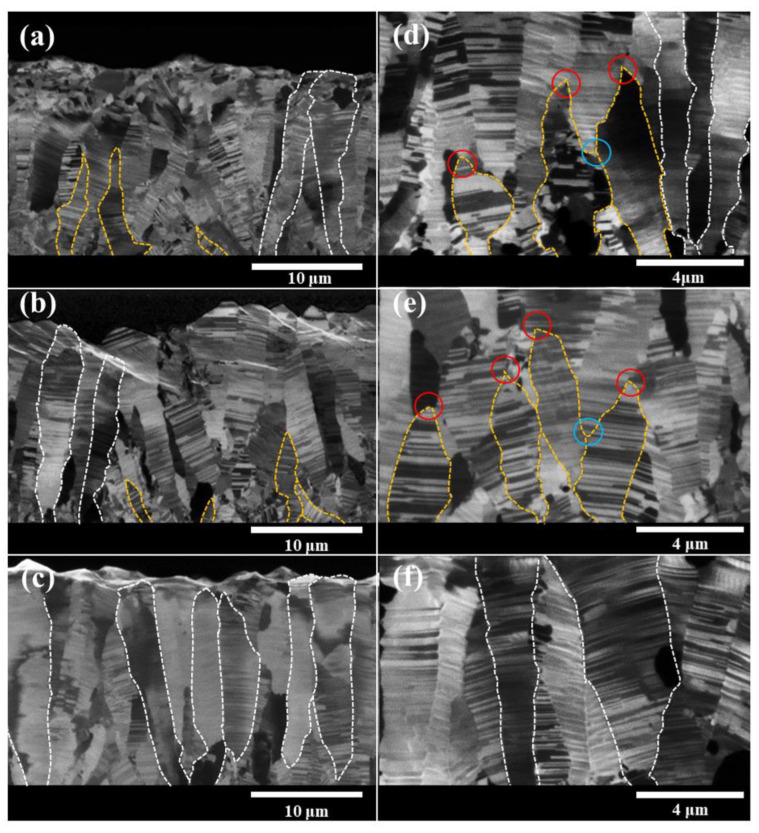
FIB cross-sectional images of the electroplated nt-Cu films. Images (**a**–**c**) are the low-magnification FIB images corresponding to the nt-Cu films electroplated with the Cu-sulfate base plating solution added with the concentration of gelatin (8 g/L), (7.9 g/L), and (7.8 g/L), respectively. Images (**d**–**f**) are the high-magnification FIB images relative to (**a**–**c**).

Figure 1 presents FIB cross-sectional images showing the microstructure of the electroplated nt-Cu films. Figure 1a–c corresponds to the nt-Cu films electroplated with the Cu-sulfate base plating solution added with the concentration of gelatin (8 g/L), (7.9 g/L), and (7.8 g/L), respectively. The mechanism of the gelatin additives for the formation of the twin structure has been proposed by Sun el al. [35]. During the Cu electroplating process, the gelatin would adsorb and desorb on the Cu surface of the cathode. The adsorption and desorption of the gelatin on the cathode Cu surface cause the alternative stress change in the plating Cu surface. The alternative stress accumulation and relaxation result in the formation of twin structure, especially for the mechanical twins. As seen in the low-magnification FIB cross-sectional images in Figure 1a–c, the columnar Cu grains could be observed in all three nt-Cu films plated with different concentrations of gelatin additives. To have a better illustration for the readers, some columnar Cu grains are delineated with the dashed lines, see Figure 1a–c. The growth direction of the columnar Cu grains is relatively normal to the nt-Cu films. Furthermore, interestingly, two different growth modes of the columnar Cu grains are found in the nt-Cu films plated with different concentrations of gelatin. As shown in Figure 1c, most of columnar Cu grains grew all the way from the substrate to the surface of the nt-Cu film (plated with the gelatin concentration of (7.8 g/L)), which are delineated with white-dashed lines and defined as all-the-way Cu columnar grains. On the other hand, besides all-the-way Cu columnar grains, few Cu columnar grains stopped growing at the middle of the nt-Cu films, which are delineated with the yellow-dash lines and defined as middle-stop Cu columnar grains.

For the nt-Cu films plated with the gelatin concentration of (7.9 g/L and 8 g/L), as shown in Figure 1a,b, all-the-way Cu columnar grains and middle-stop Cu columnar grains are roughly evenly mixed. From the high-magnification FIB images shown in Figure 1d–f, the growth of the middle-stop Cu columnar grains are gradually ending with a sharp tip, as shown with the red circles in Figure 1d,e. We would expect that new Cu columnar grains would nucleate and grow at the valley sites among the middle-stop Cu columnar grains, as seen in the blue circles in Figure 1d,e.

**Figure 2:**
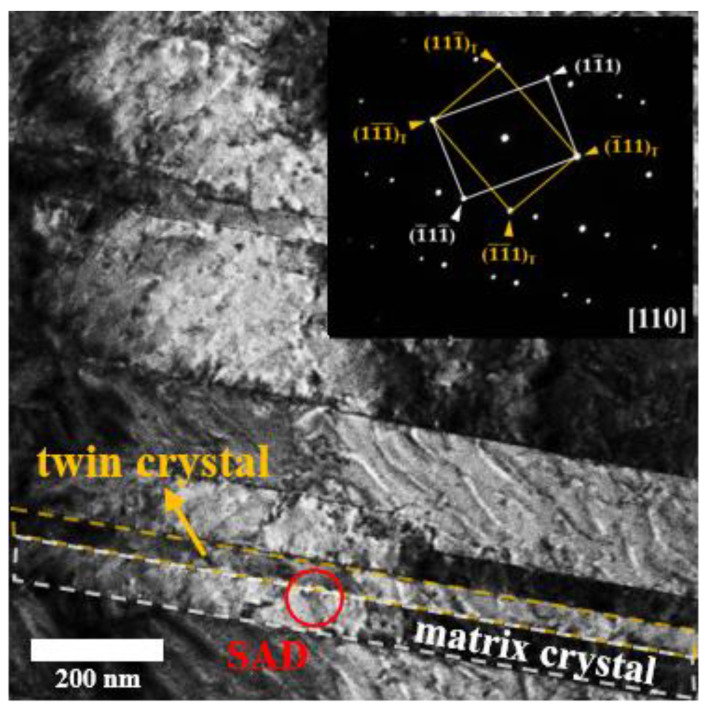
Bright-field TEM image showing the microstructure of twin grain structure of the nt-Cu films and its selected area diffraction pattern, respectively.

Figure 2 is the bright-field TEM image showing the microstructure of twin grain structure of the nt-Cu film plated with the concentration of 7.8 g/L gelatin. Clearly, the lamellar granular structure can be seen in the bright-field TEM image. In the study of twin grains, people define the lamellar grains with alternative “twin” lamellar grain and “matrix” lamellar grain [10,36,37]. As seen in Figure 2, the “twin” lamellar grain is delineated with a yellow dashed line and the “matrix” lamellar grain is delineated with a white dashed line. TEM selected area diffraction was performed at the interface (indicated with a red circle with a diameter of 100 nm). Therefore, it means that TEM selected area diffraction would include both twin lamellar grain and matrix lamellar grain. The zone axis of the selected area diffraction is <110>. As shown in the right-corner insert, i.e., TEM selected area diffraction pattern, two sets of reciprocal lattices with a mirror-symmetrical can be observed. In the selected area diffraction pattern, white-color spots belong to the matrix lamellar grain, which are corresponding to $\left(1\bar{1}1\right)$, $\left(\bar{1}11\right)$, $\left(\bar{1}1\bar{1}\right)$, and $\left(1\bar{1}\bar{1}\right)$. Moreover, the yellow-color spots belong to the twin lamellar grain, which are corresponding to $\left(11\bar{1}\right)$_T_, $\left(\bar{1}11\right)$_T_, $\left(\bar{1}\bar{1}1\right)$_T_, and $\left(1\bar{1}\bar{1}\right)$_T_. The result of TEM selected area diffraction pattern suggests that the twin grains in the present electroplated nt-Cu films grew preferentially on the {111} plane family. The coincident sites boundary index, Σ, specifies the coherence degree of the grain boundary between two grains, which is 3 for the twin boundary [38,39,40]. The lower the value of Σ, the more coherent the grain boundary. A Σ1 boundary denotes no boundary or nearly perfect boundary between crystals. Hence, the twin boundary has a relatively high coherence compared to other types of grain boundaries. Furthermore, we note that the selected area diffraction patterns of the “twin” lamellar grain and the “matrix” lamellar grain are mirror-symmetrical. This further confirms that the interface between the “twin” lamellar grain and the “matrix” lamellar grain is the twin grain boundary.

With the low-magnification FIB images shown in Figure 1a–c, the important parameters for the microstructure of the twin grains in the nt-Cu films, such as the spacing and the density of the twin boundary in the Cu columnar grains, can be calculated. The average twin spacing of the nano-twin grains is calculated to be 117.7, 131.4, and 133.7 nm for the nt-Cu films plated with the concentration of gelatin (8 g/L), (7.9 g/L), and (7.8 g/L), respectively. A total of 45 columnar grains were taken to calculate the average twin spacing. The density of the twin boundary is estimated as follows: firstly, the total length of the twin boundaries in every columnar grain (shown in Figure 1) is estimated. Furthermore, the twin-boundary density is obtained by dividing the total length of the twin boundary with the area of the columnar grain. The average twin-boundary density for the nt-Cu films plated with the concentration of gelatin (8 g/L), (7.9 g/L), and (7.8 g/L) is calculated to be, 4.3, 2.5, and 4.8 μm^−1^, respectively. The average twin-boundary density and twin spacing for the nt-Cu films plated with varying gelatin concentration are also tabulated in Table 1.

### 3.2. Microstructure Evolution of nt-Cu Films with Tensile Test

**Figure 3:**
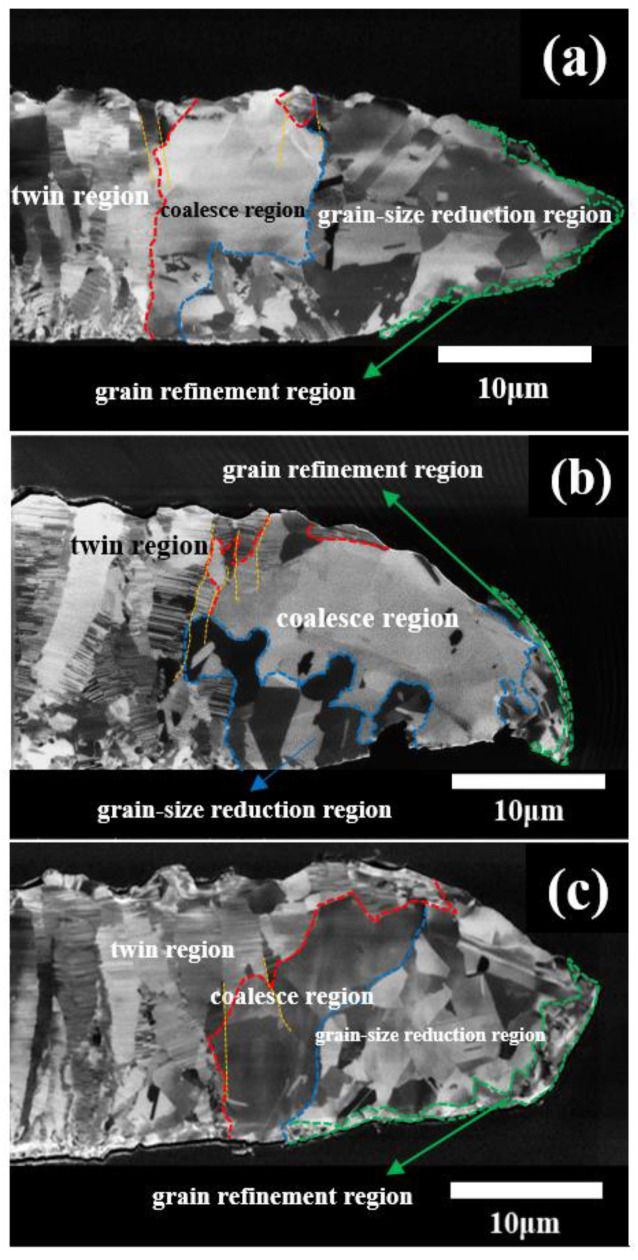
FIB images show the fractured nt-Cu films (**a**–**c**) after the tensile tests plated with the concentration of gelatin (8 g/L), (7.9 g/L), and (7.8 g/L), respectively. We clearly observed the necking phenomenon and defined the coalesce region, grain-size reduction region and grain refinement region in the necking area from images (**a**–**c**), respectively.

Figure 3a–c presents the FIB images on the fractured nt-Cu films plated with different concentrations of gelatin after the tensile tests. Clearly, we observe the necking phenomenon on the fractured nt-Cu films, which indicates a ductile fracture for the nt-Cu films. With the FIB images, the necking area is defined with the red dash lines. The length of the necking area is estimated to be about 20 μm. We can see that the columnar Cu grains still exist in the area outside the necking region. Moreover, the nano-twinned structure can be observed in the columnar Cu grains. Inside the red dash lines, as seen in Figure 3, the nano-twinned structure vanishes. This has been referred to as de-twining [34,41,42].

For the FCC metals, the {111} family planes are the slip planes, and the possible slip directions on the slip planes is <110> direction family. Thus, for the present nt-Cu films, there are 12 slip systems, as listed in Table 2. The 12 slip systems can be categorized into three types (I, II, and III), according to the relative geometrical relation between the twin boundary and the slip planes, as seen in Table 2. The slip planes of the type-I and type-II slip systems would intersect with the twin boundary with a certain angle. Thus, as the dislocations are activated to move on the slip planes by the external stress, the dislocations would eventually encounter with the twin boundaries. Consequently, dislocations would be restricted by the twin boundary. Thus, type-I and type-II slip systems are referred to as the hardening mode, which can strengthen the metals. For the type-III slip system, both the slip direction and the slip plane are parallel to the twin boundary. Hence, the dislocation movement for the type-III slip system would not encounter with the twin-boundary, which relates to the elongation to failure. The type-III slip system is called the softening mode, which promotes the elongation to failure of the nt-Cu films.

**Figure 4:**
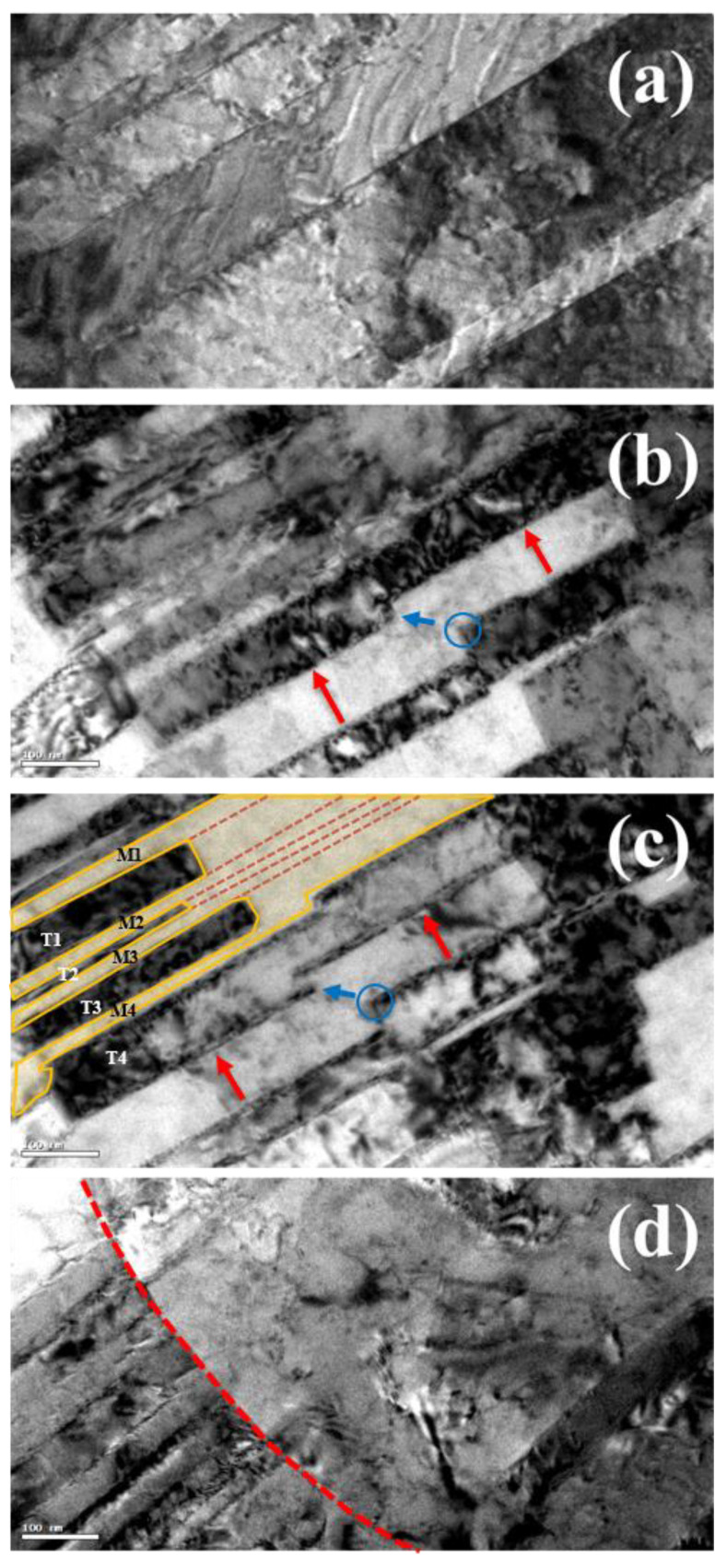
(**a**) is the initial twin structure of the nt-Cu film, (**b**) reveals a ledge occurring in the twin laminar crystal, (**c**) shows the movement of the ledge leading to the collapse between two opposite twin-boundaries, and (**d**) shows the de-twinned region.

Zhu et al. proposed a dislocation mechanism for the de-twining process [43]. They explained that, as the dislocations (type-I and type-II slip systems) move to encounter the twin boundary, the twin boundary would be converted to the Σ1 coherent boundary, which is called de-twining. In this work, the de-twining can be seen in TEM images shown in Figure 4. TEM images of Figure 4 were not taken from the same observation area. Thus, the following two-step de-twinning mechanism is inferred from the basis of the non-direct evidence. Figure 4a shows the TEM image on the initial twin structure of the nt-Cu film. Figure 4b shows the TEM images on the de-twinning region of the fractured Cu film after the tensile test. The blue circle in Figure 4b shows a ledge occurring in the twin laminar crystal in the strained nt-Cu film. It has been proposed that the ledge is formed by the accumulation of the dislocations on the twin-boundary [44,45]. Typically, those dislocations belong to type-I and type-II slip systems, which are discussed in the previous section. It can be expected that, as the dislocations continuously encounter with twin-boundaries, the under-cut depth of the ledge would increase, which leads to the movement of the twin boundary toward to the opposite twin boundary. As indicated with the red arrow, the movement of the ledge leads to the collapse between two opposite twin-boundaries, which results in the vanishing of the twin boundaries. As indicated by the blue arrow, as the under-cut corner of two ledges connects, a “cut-off” shape occurs in a laminar twin crystal, as shown by twin crystals (T1, T2, and T3), delineated with the red dashed lines shown in Figure 4c. As a result, the twin crystals gradually convert into the matrix laminar crystal, as shown with the yellow solid line in Figure 4c. Eventually, the initial twin grains in the columnar Cu grains are de-twinned and form the de-twinned region.

Thus, we conclude that the mechanism of the de-twinning attributes to two processes: (1) the ledge formation by the engagement of the dislocations with the twin boundaries and (2) the collapse of the ledges with the opposite twin-boundaries.

As shown in Figure 4d, the de-twinned region is shown in the area on the right of the red dashed line. In the region right next to the red dash line, the initial twin boundaries in the columnar Cu grains have been de-twinned, which is named as the de-twinned columnar Cu grains. By observing the orientation of the twin boundaries, i.e., (111) plane-family, in the columnar Cu grains, the orientation mismatch among the columnar Cu grains in the nt-Cu films is very minimal. Hence, under the tensile stress, the mismatched columnar Cu grains (de-twinned columnar Cu grains) could be aligned and coalesce into larger Cu grains, which is defined as the coalescence region, i.e., the region between red and blue dash lines, as seen in Figure 3a–c. In the coalescence region, the thickness reduction is very minimal, less than 3%. The thickness reduction corresponds to the degree of the plastic deformation and the cold-work percentage. Next to the coalescence region, i.e., the region between the blue dashed line and the green solid line, the size of the Cu grains is reduced, which is defined as the grain-size reduction region. Furthermore, the average thickness reduction in the grain-size reduction region is about 26%, which provides a sufficient cold-work energy to demolish the large coalesced Cu grains and the subsequent re-crystallization and regrowth processes. A very fine granular structure is found around the tip area, which is called the grain refinement region. In this grain refinement region, the Cu grains is subjected with a severe cold-work deformation.

Based on the above discussion, three regions inside the necking region are defined: coalescence region, grain-size reduction region, and grain refinement region. We proposed a microstructure evolution of the nt-Cu films in the process of the tensile test, which is described as follows. Once the nt-Cu films experience tensile stress, firstly, the de-twinning occurs in the nano-twinned columnar Cu grains. Then, the de-twined columnar Cu grains proceeds the coalescence process, which is denoted as the coalescence region (the region between the red dashed line and the blue dashed line). Next to the coalescence region, the Cu grains are subjected with a larger cold-work degree, i.e., a high thickness reduction percentage. The large coalesced Cu grains transformed to smaller Cu grains. We define this region as the grain-size reduction region, i.e., between the blue dashed line and the green solid line. In the very tip on the fracturing necking tips, Cu grains are crashed into very fine Cu grains by a severe cold-work deformation, which is defined as the grain refinement region.

### 3.3. Tensile Tests of nt-Cu Films with Different Twin-Boundary Density

**Figure 5:**
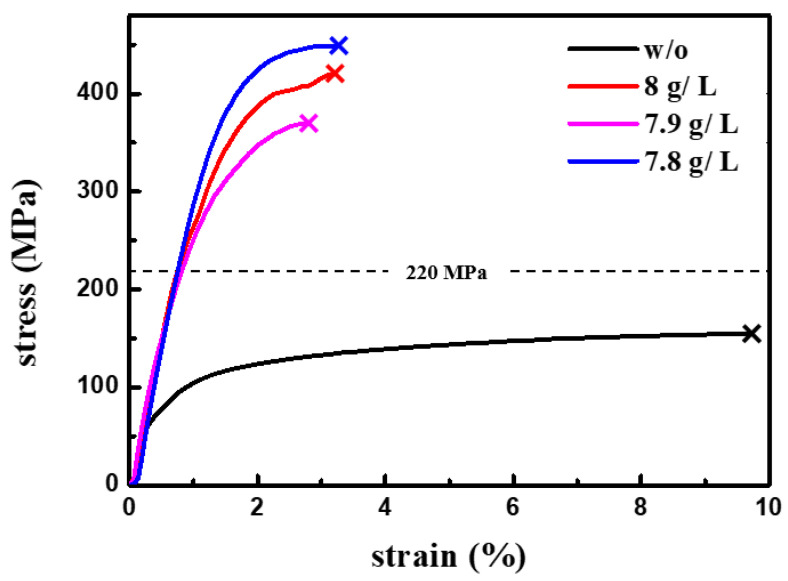
Stress–strain curves of the nt-Cu films electroplated with the Cu-sulfate base plating solution added with the concentration of gelatin (0 g/L), (8 g/L), (7.9 g/L), and (7.8 g/L), respectively.

Figure 5 shows the typical stress–strain curves of the nt-Cu films plated with different concentration of gelatin additives. With stress–strain curves in Figure 5, the Young’s modulus of the nt-Cu films plated with the gelatin concentration of (8 g/L), (7.9 g/L), and (7.8 g/L) can be determined to be 315.4, 308.6, and 318.9 MPa, respectively. Moreover, the yield strength of the nt-Cu films plated with the concentration of gelatin (8 g/L), (7.9 g/L), and (7.8 g/L) is 274.3, 256.5, and 347.9 MPa, respectively. All three nt-Cu films break at their maximum strength. Thus, instead of fracture strength, we take the maximum strength as the fracture strength. The fracture strength of the nt-Cu films plated with the gelatin concentration of (8 g/L), (7.9 g/L), and (7.8 g/L) is 420, 369, and 449 MPa, respectively. The elongation to failure of the nt-Cu films plated with the gelatin concentration of (8 g/L), (7.9 g/L), and (7.8 g/L) is 3.1%, 2.7%, and 3.2%, respectively. Three Cu films were prepared at each plating condition, in terms of the gelatin concentration. Thus, three mechanical parameters would be obtained for the Cu film plated with each gelatin concentration. The mechanical parameters tabulated in Table 3 are the average value from three testing results. For the comparison purpose, we also prepared a Cu film plated without adding gelatin. The microstructure of the Cu film plated without gelatin is the typical thin inclined columnar structure. We also note that no nano-twin structure formed in the Cu film plated without adding gelatin. The black curve in Figure 5 shows the stress–strain curve of the Cu film plated without adding gelatin. The fracture strength of the Cu film plated without adding gelatin is about 150 MPa and the elongation to fracture is about 10 %. The Cu film electroplated with no gelatin additive has the much lesser strength than the nt-Cu films plated with the gelatin. However, the Cu film electroplated with no gelatin additive has a larger elongation to fracture than other nt-Cu films. In addition, the observation of necking in the Figure 3 implies that there should be a corresponding region of softening in the stress–strain curves in Figure 5. However, in fact, no corresponding regions of softening can be found in the stress–strain curves in Figure 5. At this point, we speculate that the necking happened in a very short time, because the necking region is only about 20 μm. Thus, the softening behavior due to the necking is very minimal, which is not clearly shown in the stress–strain curves in Figure 5.

The linear curve in the stress–strain curves represents the elastic deformation region. We found that the slope of the linear curves in the stress–strain curves of the nt-Cu films electroplated with three different concentrations of gelatin additives are about the same. It means that Young’s modulus of the nt-Cu films electroplated with three different concentrations of gelatin additives are similar. As the stress is about 220 MPa, the stress–strain curves of the nt-Cu films start deviating from the linear relation. The deviation from the linear curve is the indicator of the starting of the plastic deformation. Generally, the plastic deformation is mainly resulted from the completion of the dislocation movement. The completion of the dislocation movement means that the dislocation inside the nt-Cu films moves and reaches the outer surface to make an actual displacement of the metals. In the present nt-Cu films, the dislocation movement is prone to be retarded by twin-boundaries within a very small moving distance, due to the small twin spacing in nano scale. The completion of the dislocation movement is only possible if the twin-boundaries have been unhindered. Hence, the completion of the dislocation movement to make plastic deformation possible requires that it (1) activates the dislocation movement in the nt-Cu films, and also, (2) clears the potential restriction by the twin-boundaries, i.e., de-twining.

As shown in Figure 5, the on-set of the plastic deformation of the nt-Cu films against the stress level is about the same, around 220 MPa. It implies that the stress level of 220 MPa is ample to activate the dislocation movement in the nt-Cu films and de-twin the twin structure in nt-Cu films. Moreover, we found that, as the stress over 220 MPa, the deviation degree from the linear curves against the stress level varies with the density of the twin boundary in the nt-Cu films. The deviation degree from the linear curves is inversely proportional to the density of twin boundary in the stressed nt-Cu films, which corresponds to the plastic displacement (i.e., de-twining) of the nt-Cu films. The above finding means that (1) the de-twining starts at the stress level about 220 MPa and (2) the de-twining percentage is inversely proportional to the density of twin boundary in the stressed nt-Cu films as the external stress over 220 MPa. Thus, a higher stress level is required to complete the de-twinning process, i.e., clearing a twin-boundary-free path for the dislocation movement in the stressed nt-Cu films with a larger twin-boundary density. Based on the above observation and discussion, the fracture strength of the nt-Cu films would be governed by the completion of the de-twinning process, which depends on the twin-boundaries density. Hence, as shown in Table 1 and Table 3, the strength of the nt-Cu films is highly related to the twin-boundary density. The nt-Cu film with a greater twin-boundary density has a larger fracture strength than the nt-Cu film with a lesser twin-boundary density.

## 4. Conclusions

Cu columnar grains with nano-twin structure can be produced in the nt-Cu films electroplated with the Cu-sulfate electrolyte solution added with varying gelatin concentration. With TEM image and selected area diffraction pattern, nano-twinned structure can be observed and defined in the electroplated Cu films. The average twin-boundary density and twin spacing for the nt-Cu films plated with the concentration of gelatin additive (8 g/L), (7.9 g/L), and (7.8 g/L) are calculated with TEM images, which are found to depends on the concentration of gelatin additive. For the FCC Cu films, there are 12 slip systems, which are categorized into three types (I, II, and III), according to the relative geometrical relation between the twin boundary and the slip planes. Thus, type-I and type-II slip systems are referred as the hardening mode, which can strengthen the metals. For the type-III slip system, both the slip direction and the slip plane are parallel to the twin boundary. Hence, the dislocation movement for type-III slip system would not encounter with the twin-boundary, which relates to the elongation to failure. Once the Cu films experience tensile stress, firstly, the de-twinning occurs in the nano-twinned columnar Cu grains. Then, the de-twined columnar Cu grains proceed the plastic deformation. Thus, the plastic deformation requires that it (1) activates the dislocation movement in the nt-Cu films, and also, (2) clears the potential restriction by the twin-boundaries, i.e., de-twining. We conclude that the plastic deformation is governed by the de-twinning for the nano-twinned columnar Cu grains, which depends on the twin-boundaries density. Hence, we conclude that the strength of the nt-Cu films is highly related to the twin-boundary density. The nt-Cu film with a greater twin-boundary density has a larger fracture strength than the nt-Cu film with lesser twin-boundary density. The two-step de-twinning mechanism and the microstructure evolution process in the tensile test of the nt-Cu films proposed in the conclusions is limited in the Cu films, which has the columnar grain structure

## Figures and Tables

**Table 1:** The average twin-boundary density and twin spacing for the nt-Cu films plated with the concentration of gelatin (8 g/L), (7.9 g/L), and (7.8 g/L).

Concentration of Gelation	8 g/L	7.9 g/L	7.8 g/L
Twin Spacing (nm)	117.7	131.4	133.7
TB Density (1/μm)	4.3	2.5	4.8

**Table 2:** A total of 12 slip systems for the present nt-Cu films.

Type	Slip System
Type I	$\left(1\bar{1}1\right)\left[\bar{1}01\right]$
	$\left(\bar{1}11\right)\left[01\bar{1}\right]$
	$\left(1\bar{1}1\right)\left[\bar{1}01\right]$
Type 2	$\left(1\bar{1}1\right)\left[011\right]$
	$\left(1\bar{1}1\right)\left[110\right]$
	$\left(\bar{1}11\right)\left[101\right]$
	$\left(1\bar{1}1\right)\left[110\right]$
	$\left(11\bar{1}\right)\left[101\right]$
	$\left(11\bar{1}\right)\left[011\right]$
Type 3	$\left(111\right)\left[01\bar{1}\right]$
	$\left(111\right)\left[\bar{1}10\right]$
	$\left(111\right)\left[\bar{1}01\right]$

**Table 3:** The Young’s modulus, yield strength, fracture strength, and elongation to failure for the nt-Cu films plated with the concentration of gelatin (8 g/L), (7.9 g/L), and (7.8 g/L).

Concentration of Gelatin	8 g/L	7.9 g/L	7.8 g/L
Young’s Modulus (MPa)	315.4 ± 15	308.6 ± 9	318.9 ± 16
Yield Strength (MPa)	274.3 ± 13	256.5 ± 7	347.9 ± 17
Fracture Strength (MPa)	420 ± 20	369 ± 10	449 ± 23
Elongation to Failure (%)	3.1 ± 0.1	2.7 ± 0.1	3.2 ± 0.1

## Data Availability

The raw/processed data required to reproduce these findings cannot be shared at this time as the data also forms part of an ongoing study.
